# Foundation doctors in Anaesthesia: should they be taught to administer an anaesthetic?

**DOI:** 10.1186/1472-6920-7-48

**Published:** 2007-11-28

**Authors:** Alexander W Phillips, Abhinav Kant, James P Chinery, Sean Williamson, David M Murray

**Affiliations:** 1 Specialty Trainee 2, Cardiothoracic Surgery, John Radcliffe Hospital Headley Way, Headington, Oxford, OX3 9DU, UK; 2 Specialty Trainee 3, Bradford Royal Infirmary, Duckworth Lane, Bradford, West Yorkshire, BD9 6RJ, UK; 3 Specialty Trainee 1, James Cook University Hospital, Marton Road, Middelsbrough, TS4 3BW, UK; 4 Consultant Anaesthetist, Cleveland School of Anaesthesia, James Cook University Hospital, Marton Road, Middlesbrough, TS4 3BW, UK

## Abstract

**Background:**

Anaesthetic pre-registration house officer posts have been available since 1997. With the change to postgraduate medical training introduced in 2005, these posts have become vital building blocks for Foundation Programmes.

**Discussion:**

We debate the skills that new Foundation Programme doctors in such posts should be taught, particularly whether administration of an anaesthetic holds an important place. The opinion of college tutors prior to the institution of the foundation programme is included. These were obtained from a postal questionnaire.

**Summary:**

We maintain that teaching how to administer an anaesthetic remains an important learning objective and something that should be actively pursued.

## Background

**Figure 1:**
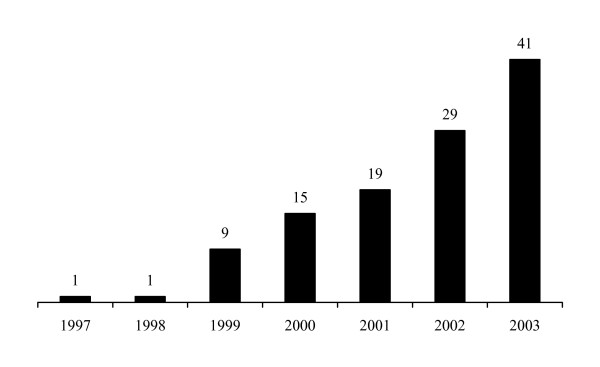
Number of Trusts offering anaesthetic pre-registration house officer posts each year 1997–2003.

The traditional idea of pre-registration doctors undertaking two 6-month house jobs, one in medicine and one in surgery, often in different hospitals, has been superseded. Since 1997 there has been an increase in pre-registration rotations, which have included specialities such as paediatrics, emergency medicine, obstetrics and gynaecology and anaesthesia with critical care. Much of this stemmed from the publication of The New Doctor in 1997 [1], which promoted these 4-month jobs involving an alternative clinical speciality and gave specific and comprehensive objectives for pre-registration house doctors to achieve whilst carrying out their role as a junior doctor. The inclusion of anaesthetics in these pre-registration years has seen substantial growth over the years 1997–2003 (Figure 1).

The service role for medical and surgical pre-registration house officers was well defined by the nature of ward and theatre work. The situation is less clear in anaesthesia as the clinical role for pre-registration house officers and now Foundation House officers are undefined. Even new-starter specialty trainees do not undertake a service commitment until they have completed their initial assessment of competency (normally performed after three months) allowing them to work under indirect supervision and take part in on-call commitments [2]. The ability of a pre-registration house doctors to undertake a service role is therefore limited by the need for an intensive initial period of training that lasts almost as long as the post itself. This may create the impression for trainers that there is little motivation or value in teaching pre-registration doctors how to give an anaesthetic.

Previously, Pre-Registration House Officers (PRHOs) in anaesthetics had a supernumerary role which allowed them to receive one-to-one teaching and supervision from a consultant anaesthetist. Time spent in theatre would allow skills in airway management, and line insertion through to being able to administer an anaesthetic to be developed. Often a period of the attachment would be spent in an ITU/HDU setting allowing PRHOs to become familiar with patient treatment at this level and skills in recognising and evaluating critically ill patients. Service commitment was usually made up by taking part in either the surgical or medical on-call rotas.

We debate the importance of being able to administer an anaesthetic. We briefly include own data collected in the final years prior to the institution of the foundation programme as to what college tutors at hospitals incorporating PRHO anaesthetic posts regarded as important.

## Discussion

### Implementation of posts

Since the initiative to amend the Medical Act in 1997 allowing a change in the first postgraduate year's training, there has been a substantial increase in placements outside the conventional medicine and surgery posts [3]. The expansion of posts offering anaesthesia, which will be filled with doctors who do not have an intention to pursue anaesthesia as a career is set to increase with the introduction of the Foundation programme [4]. Further the decoupling of Foundation 1 and Foundation 2 years as suggested by the Tooke report [5] will probably result in a system similar to that prior to 2005. At present, even with the introduction of run-through training under Modernising Medical Careers, there is little specific guidance as to the content of these training posts or how learning objectives should be assessed under a standardised national framework. As a result, individual Trusts have been left to interpret the needs of anaesthetic pre-registration doctors in their own way and are likely to have implemented training programmes that vary widely in both structure and content. Despite the changes to medical training brought about by Modernising Medical Careers, the learning needs of doctors within Foundation posts is very similar to that of pre-registration house officers. It is likely that existing pre-registration house officer posts were used as a template for the new Foundation posts and will be used as a basis in any forthcoming changes to the structure of junior doctor training that may result as a consequence of Tooke's recommendations. We feel that the success of Foundation posts to fulfil their desired objectives may be limited by this lack of congruity in the training provided between these posts throughout the country.

The General Medical Council requires that pre-registration house officer posts have in place a formal mechanism of assessment and a formal educational programme [6]. Virtually all Trusts have this in place, although the format varies in each Trust. However there is greater variation with regard to the specific skills that Pre-registration house officers are taught.

### Core Skills learned from giving an anaesthetic

There are various skills that anaesthetists can impart to a house officer, which may be of use for their future career. Vital clinical skills including intravenous cannulation, administering intravenous drugs, bag and mask ventilation, fluid management and patient monitoring, are routinely used on all wards but with varying levels of proficiency and underlying knowledge [7]. Stewart et al [8] identified core skills, attitudes and knowledge which a PRHO should acquire. These were in similar to the opinions of anaesthetic clinical tutors responsible for PRHOs (Table 1). Those that are not considered to be core skills required by house officers on a ward, such as obtaining central vascular and arterial access, receive correspondingly less emphasis.

**Table 1:** Importance of tasks for pre-registration house officers.

Task	Number (proportion) (n = 43)
Bag & Mask ventilation	43 (100)
Fluid Management	43 (100)
Intravenous Cannulation	43 (100)
Perioperative analgesia	43 (100)
Recognise Critical illness	43 (100)
Preparation and administration of intravenous drugs	42 (98)
Acute Pharmacology & Physiology	38 (88)
Patient monitoring & documentation	38 (88)
Preoperative assessment	34 (79)
Laryngeal Mask Airway insertion	29 (67)
Use of local anaesthetic	28 (65)
ALS Course completion	26 (60)
Endotracheal intubation	25 (58)
Maintaining a log book of experience	23 (53)
Central venous line insertion	22 (51)
Arterial line insertion	18 (42)
Anaesthetic machine check	7 (16)

Tasks considered by respondents to be either essential or very valuable learning objectives for pre-registration house officers to achieve. Values are number of respondents (% of the 43 respondents).

The nature of anaesthesia and critical care makes these posts an excellent opportunity for training junior doctors to recognise critical illness including an understanding of the underlying physiology and pharmacology. Many of the competencies considered important to be gained in an anaesthetic post are those that might be considered generic for all doctors. Those tasks that may be regarded as less important for pre-registration house doctors in anaesthetics are those pertaining to anaesthesia such as checking the anaesthetic machine, intubation and laryngeal mask insertion. This may seem surprising given that these are inherent skills for an anaesthetist, but may reflect the fact that pre-registration house officer posts were not intended to provide the doctor with an ability to anaesthetise patients. Our survey of clinical tutors supported this position (Table 2), however we feel that giving an anaesthetic should remain an important objective.

**Table 2:** Should pre-registration house officers be taught to administer an anaesthetic?

	Induction	Induction & Maintenance
Never	7(16)	11 (26)
Yes, in all cases	8 (19)	5 (12)
Yes, depending on ability	28 (65)	27 (63)
Supervised from same room	29 (67)	26 (60)
Supervised from corridor	5 (12)	4 (9)
Supervised from coffee room	2 (5)	2 (5)

Consultant opinion regarding whether pre-registration house officers should be allowed to induce anaesthesia, or induce and maintain anaesthesia, and with what degree of supervision. Values are number of respondents (% of the 43 respondents) who gave each opinion.

### The question of giving an anaesthetic

Whether or not a consultant anaesthetist would teach a foundation doctor how to "give an anaesthetic" is likely to depend on the trainee's perceived ability. However it is likely that this trainee group will vary both in motivation and experience partly dependent on progress throughout the pre-registration year. Observationally pre-registration house doctors can find an anaesthetic attachment a potential extension of medical school with the benefit of a salary, a view possibly potentiated by the lack of an obvious service role. It therefore falls to the consultant anaesthetist to convey the importance of achieving this as a learning objective during their anaesthetic attachment in order that the trainee appreciates the reasons for their needing to administer an anaesthetic, and develops the motivation to do so. We feel that more specific guidance would be valuable in order to improve the educational value of these posts. Without this, the ability of the trainee to be fully competent in the tasks identified as important for the pre-registration doctor may be limited for the reasons discussed below.

Skills such as maintaining an airway or prescription of post-operative analgesia, whilst important in themselves, are not carried out as isolated tasks, but as part of a clinical situation requiring the correct performance of all those tasks in order to bring about the satisfactory outcome for a patient. The action of giving an anaesthetic focuses the mind, and brings together individual skills into a complex action that can be observed by the consultant. In this way, the trainee demonstrates that they can actually perform the task in practice, rather than demonstrating that they merely have the knowledge to be able to perform that particular task [9,10]. Ultimate responsibility will always rest with the supervisor but a learner must be able to develop the attitude of being responsible for the patient, a point that is recognised in The New Doctor [6]. Allowing foundation doctor to conduct all parts of an anaesthetic creates that sense of responsibility for patient. The ability to make a decision and reflect on the correctness or otherwise of that decision is a fundamental difference between the medical student and the new doctor. This sense of responsibility also creates a learning need for the pre-registration house officer.

### Improved learning if there is a service role

Adults are more likely to learn if the task is relevant to the demands of their everyday life [11]. The need to take responsibility for the patient creates an immediate relevant learning need for the trainee and therefore focuses their mind towards learning that skill. Olympio [12] used a simulator to successfully teach trainee anaesthetists a new technique to manage oesophageal intubation. When subsequently tested in a different scenario that contained an oesophageal intubation, the trainees failed to utilise the new technique. An accompanying editorial [13] felt that one factor responsible for this failure was that the need to learn this new skill was not relevant to the trainees' current learning needs. They were already capable of recognising and managing an oesophageal intubation, and did not perceive the need to learn a new technique. Skills such as airway management may be taught on a manikin, but are not done so as the value of being able to manage an airway in real life is recognised. In our view it would be unfortunate if the house officer were not given an opportunity to develop skills during the management of real cases. In not using all the opportunities that anaesthetising real patients presents to the learner, we are in danger of not realising the full potential of an anaesthetic pre-registration house officer post. As our results demonstrate, whilst many consultants may allow the pre-registration house officer to conduct an anaesthetic, this was dependent on their perceived ability to do so.

### Supervising such junior trainees without formally recognised competencies

There are clear guidelines from the Royal College of Anaesthetists which state the level of supervision that must be provided for a trainee who has yet to achieve their "New Starter" initial assessment competency [2]. Prior to achieving these competencies, a doctor is unable to practice anaesthesia at any time without direct supervision. In our own hospital, if a trainee shows appropriate ability, then they are able to achieve these competencies as a foundation doctor, although this does not negate the need for that trainee to obtain these competencies if they commence a formal specialist training post in anaesthesia.

This progressive release of control mirrors the necessary process that must occur with new starter specialist trainees in order to reach the initial test of competency and practise anaesthesia without immediate supervision. Again, this potential to work with less direct supervision creates a relevant learning need for the trainee. Demonstrating an ability to achieve preset competencies is beneficial both to those trainees seeking a career in anaesthetics and those with other career aspirations. Despite this it would be prudent to suggest that at this junior level, with only limited experience as a doctor (some may be working in their very first job and have no more than a few weeks experience) close supervision at all times would be appropriate. We feel supervision from the anaesthetic room would be most fitting. The level of input required from the consultant, may however be negligible.

### Represented Views

The response rate to our survey was high (86%), and responses obtained from a wide geographical area. It is unlikely that the attitudes of college tutors will have changed drastically with the implementation of the foundation programme. Further, the recommendation of the Tooke report is a decoupling of the foundation 1 and foundation 2 years. This will result in a training scheme not dissimilar to that in place prior the institution of Modernising Medical Careers (MMC), with juniors undertaking a pre-registration year followed by three years in core specialty training [5].

However, it is possible that our findings do not fully represent the views of all those directly involved in teaching pre-registration doctors. During the pilot period of the questionnaire in our own department, we found differing results in that 50% of consultants felt it inappropriate to teach a pre-registration house officer how to administer an anaesthetic. This contrasts to the results from the developed questionnaire, in which 16% felt it inappropriate. The views obtained are those of the educational supervisors and may not accurately reflect the practice of other consultants within a department also involved in educating these doctors. Whilst it is likely that the educational supervisor would have been responsible for developing the pre-registration house officer training programme within his or her department, it may be that there is a disparity between the intentions of a training programme, and what is actually delivered in theatre by individual consultants within a department. College tutors may have an idealistic view of the skills they would like juniors to develop. The tutors may also have a larger interest in teaching skewing their opinion with respect to whether administrating an anaesthetic should be encouraged in such junior doctors who may not even have a long-term interest in anaesthetics as a career. Other anaesthetic consultants may be less keen to encourage such independence and responsibility. A happy and agreed upon standard thus needs to be established for all those involved in teaching and reinforces a need for greater clarification of the role of foundation doctors in anaesthesia.

### A lack of motivation to teach

The reluctance to teach pre-registration doctors how to perform all aspects required to administer an anaesthetic is understandable. These doctors are only in post for a few months, they may not ultimately have career intentions towards anaesthesia, and the time is not recognised towards future training. This is an issue that is not confined to Foundation years. Higher specialist trainees in Accident and Emergency may also spend three months in anaesthesia as part of their own CCST training requirements with the expectation that they are "a fully integrated member of the team [14]." We are concerned that there may be a lack of direction in the training of this group of doctors, which is exacerbated by the absence of an obvious service role. As a speciality we are well versed in teaching trainees how to anaesthetise patients as this forms part of our everyday practice. There are valid educational reasons why non-specialist trainees should be taught the whole task of providing anaesthesia for fit healthy patients. Whilst foundation doctors will never be able to provide a service based role of more senior trainees, the objective of administering an anaesthetic would define their role more specifically, give greater direction to their education, and provide a greater chance of fulfilling the aims of Foundation Programmes.

## Summary

Foundation posts in anaesthetics will provide a valuable opportunity for junior doctors to develop important skills.

We feel the skill in giving an anaesthetic should not be overlooked as a target as this brings together previously learnt skills and helps focus the junior doctor.

## Competing interests

The author(s) declare that they have no competing interests.

## Authors' contributions

AWP, AK, JPC and SW conceived the original study. AWP, AK and JPC were involved in collecting data from the college tutors and collating results. AWP, AK, JPC, SW and DMM were involved in writing the initial drafts. AWP and AK were involved in the revised drafts. All authors read and approved the final manuscript.

## Pre-publication history

The pre-publication history for this paper can be accessed here:


